# Effects of demand-side financing on utilisation, experiences and outcomes of maternity care in low- and middle-income countries: a systematic review

**DOI:** 10.1186/1471-2393-14-30

**Published:** 2014-01-17

**Authors:** Susan F Murray, Benjamin M Hunter, Ramila Bisht, Tim Ensor, Debra Bick

**Affiliations:** 1 King’s International Development Institute, King’s College London, London, UK; 2 Centre of Social Medicine and Community Health, Jawaharlal Nehru University, New Delhi, India; 3 Nuffield Centre for International Health and Development, University of Leeds, Leeds, UK; 4 Florence Nightingale School of Nursing and Midwifery, King’s College London, London, UK

**Keywords:** Demand-side financing, Maternal health, Vouchers, Cash transfers, Narrative synthesis, Qualitative, Systematic review

## Abstract

**Background:**

Demand-side financing, where funds for specific services are channelled through, or to, prospective users, is now employed in health and education sectors in many low- and middle-income countries. This systematic review aimed to critically examine the evidence on application of this approach to promote maternal health in these settings. Five modes were considered: unconditional cash transfers, conditional cash transfers, short-term payments to offset costs of accessing maternity services, vouchers for maternity services, and vouchers for merit goods. We sought to assess the effects of these interventions on utilisation of maternity services and on maternal health outcomes and infant health, the situation of underprivileged women and the healthcare system.

**Methods:**

The protocol aimed for collection and synthesis of a broad range of evidence from quantitative, qualitative and economic studies. Nineteen health and social policy databases, seven unpublished research databases and 27 websites were searched; with additional searches of Indian journals and websites. Studies were included if they examined demand-side financing interventions to increase consumption of services or goods intended to impact on maternal health, and met relevant quality criteria. Quality assessment, data extraction and analysis used Joanna Briggs Institute standardised tools and software. Outcomes of interest included maternal and infant mortality and morbidity, service utilisation, factors required for successful implementation, recipient and provider experiences, ethical issues, and cost-effectiveness. Findings on Effectiveness, Feasibility, Appropriateness and Meaningfulness were presented by narrative synthesis.

**Results:**

Thirty-three quantitative studies, 46 qualitative studies, and four economic studies from 17 countries met the inclusion criteria. Evidence on unconditional cash transfers was scanty*.* Other demand-side financing modes were found to increase utilisation of maternal healthcare in the index pregnancy or uptake of related merit goods. Evidence of effects on maternal and infant mortality and morbidity outcomes was insufficient. Important implementation aspects include targeting and eligibility criteria, monitoring, respectful treatment of beneficiaries, suitable incentives for providers, quality of care and affordable referral systems.

**Conclusions:**

Demand-side financing schemes can increase utilisation of maternity services, but attention must be paid to supply-side conditions, the fine-grain of implementation and sustainability. Comparative studies and research on health impact and cost-effectiveness are required.

## Background

Despite recently renewed focus to meet the impending deadline of the Millennium Development Goals, investments have not yet achieved sustainable comprehensive public sector maternal health programmes in many countries [1]. Even where services are reasonably adequate, demand sometimes remains low [2]. When healthcare is accessed, transport and treatment costs and loss of earnings may cause poor families to descend further into poverty [1,3,4]. 'Demand-side’ financing (DSF) approaches have been seen as means to ameliorate this situation, and have been employed in many different contexts in low-and middle-income countries in attempts to help overcome barriers to access to maternity care.

Originating in the education sector, DSF refers to a group of mechanisms for 'transferring purchasing power to specified groups for the purchase of defined goods or services’ [5]. It is typically used to supplement traditional forms of 'supply-side’ financing of services which channel payments directly to service providers. In maternal healthcare programmes, DSF mechanisms have often taken the form either of vouchers that can be exchanged for subsidised goods or specific services [6-8], or of short-term cash incentives or reimbursements that are linked to service use [9,10]. However, broader social development programmes designed primarily to improve child health and education may also include regular payments to households that are in part conditional on women’s uptake of specified maternity services [11-13]. Finally, some cash transfers, provided as maternity benefits or allowances, do not impose conditions of uptake of specific goods or services but are assumed to facilitate poor women’s access to them by reducing financial barriers [14].

Three previous systematic reviews of evidence on DSF had included information on its application in maternal health [15-17], but each was confined to consideration of evidence on effectiveness of a single mode - vouchers [15,17] or cash transfers [16]. In this new systematic review, commissioned by the Australian Agency for International Development (AusAID) through the '3ie’ International Initiative for Impact Evaluation, we focused exclusively on maternal health and broadened the questions of interest to include the four facets of enquiry used in the Joanna Briggs Institute approach to systematic reviews of healthcare interventions – 'effectiveness’, 'feasibility’, 'appropriateness’ and 'meaningfulness’ [18]. This approach was chosen because it enabled us to consider a range of effects and to collate operational experiences from the implementation of schemes in different settings, as well as to consider some of the wider implications of using conditional forms of DSF in women’s healthcare. We considered quantitative and qualitative evidence on the following modes of DSF used to promote maternal health in low- and middle-income countries [19]:

• conditional cash transfers (CCT) targeted at poor households which meet various conditionalities to receive payments;

• short-term cash payments to offset costs of accessing maternity services;

• vouchers exchanged for maternity services, for which providers are later reimbursed;

• vouchers for 'merit’ goods such as insecticide-treated bed nets that promote maternal and infant health, and

• unconditional cash transfers (maternity benefits or allowances).

The overall systematic review objective was to assess the effects of DSF interventions on utilisation of maternity services and maternal health outcomes in low- and middle-income countries. Secondary outcomes included effects on maternal and infant health, the situation of underprivileged women and the healthcare system. In this article the review is reported in accordance with the PRISMA statement [20].

## Methods

The mixed-method systematic review protocol was registered with the Joanna Briggs Institute (JBI, registration number 000592). The population of interest were poor, rural or socially excluded women in low- and middle-income countries who were pregnant or within 42 days of the end of pregnancy [21]. Interventions of interest were programmes incorporating DSF as a mechanism to increase consumption of services or goods intended to impact on maternal health. As per JBI criteria, outcomes of interests were:

• Quantitative – maternal (antenatal, intrapartum and postnatal) and infant (perinatal, neonatal and infant) mortality and morbidity, and utilisation of maternity services

• Qualitative – barriers to and preconditions for successful implementation, experiences of providers, ethical issues and social meaning for women

• Economic – cost and cost-effectiveness of DSF interventions

The review’s 15 questions can be found in the full protocol (available at http://connect.jbiconnectplus.org/ViewSourceFile.aspx?0=6151) and on the PROSPERO website (registration number CRD42012002056). Studies were eligible if published between January 1990 and June 2012, and conducted in countries classified as low- or middle-income at time of study [22].

Systematic searches were conducted using 30 terms: abortion, antenatal, birth, cash transfer, child benefit, cost, cost-effective, cost-utility, demand-side financing, demand side financing, family allowance, food stamps, health service utilisation, incentive, infant, maternal, maternity allowance, maternity benefit, midwifery, monetary transfer, neonatal, morbidity, mortality, obstetric, output-based aid, perinatal, postnatal, pregnancy, reimbursement mechanism and voucher. These terms were used in 19 medical, health and social policy databases and seven databases of unpublished research (Table 1) [19]. Eighteen studies were obtained directly from authors. Additional literature was sought from websites of international organisations. As India was known for its range of small and large-scale DSF schemes for maternal health, and previous experience indicated that much Indian research and evaluation is to be found outside of the standard bibliographic databases [23], supplementary searches examined India-specific websites and journal catalogues.

**Table 1:** Search details

**Sample search strategy for SCOPUS**	**List of databases and e-journals searched**
1. (“child benefit” or “demand side financing” or “demand-side financing” or “family allowance” OR “food stamp” or “maternity allowance” or “maternity benefit”)	Applied Social Sciences Index and Abstracts, ArticleFirst, British Development Library Services, CINAHL, Cochrane Central Register of Controlled Trials, EconLit Electronic Collections Online, HealthSource: Nursing/Academic Edition, International Bibliography of the Social Sciences, Latin American and Caribbean Health Sciences, Sage Journals Online, ScienceDirect, SCOPUS, Social Policy and Practice, Social Services Abstracts, Sociological Abstracts, SpringerLink, Web of Knowledge [including Medline], Wiley Online Library
2. (“cash transfer” or “monetary transfer” or “output-based aid” or “reimbursement mechanism” or “voucher” or “incentive”)	
3. (“abortion” or “antenatal” or “birth” or “infant” or “matern$” or “midwi$” or “neonat$” or “obstetric” or “perinatal” or “postnatal” or “pregnan$”).ti,ab	
4. (“cost” or “cost-effectiv$” or “cost-utility” or “health service utili$” or “morbidity” or “mortality”).ti,ab	
5. 3 or 4	
6. 1 and 5	
7. 2 and 3	
8. 6 or 7 [Limit to: Publication Year 1990 – 2012]	

Primary (BH) and secondary reviewers (SFM, DB) independently reviewed all papers and conferred. Studies were assessed for methodological quality. Data extracted using JBI’s standardised tools included: author/year, participants, intervention, setting, sample size, risk of bias, and outcomes of significance including odds ratios, marginal effects or differences in means, and levels of statistical significance, if presented. As meta-analysis of quantitative data was not possible due to study heterogeneity, quantitative and economic findings are presented in narrative form. Qualitative findings were categorised thematically as the iterative analysis progressed and were subjected to meta-synthesis using JBI software [24]. Synthesised findings were grouped into topic areas under the relevant dimension (feasibility, appropriateness, meaningfulness) as presented in this paper. The review did not include any questions to examine any effect of DSF on purchasing power, such as disposable income or buying habits.

## Results

**Figure 1:**
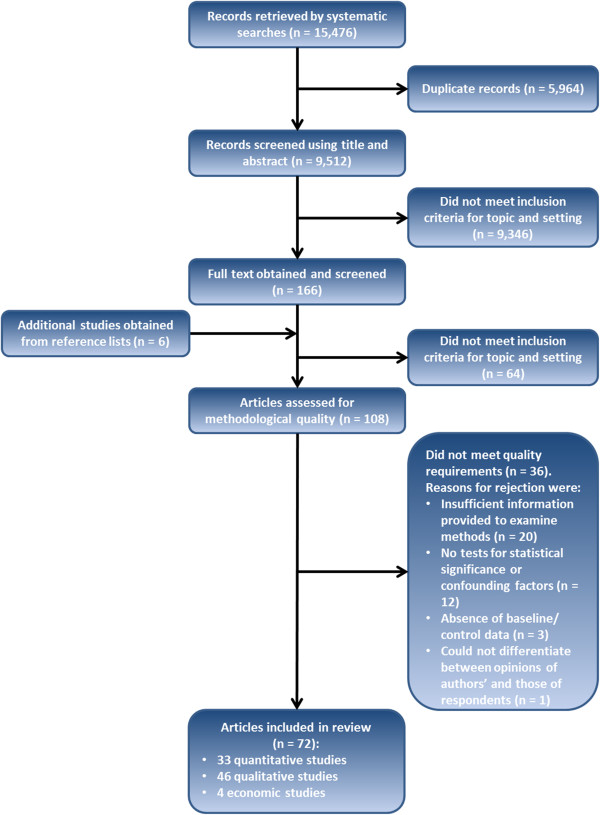
Flowchart of study selection.

Figure 1 presents the search, screening and assessment processes. The final selection included 72 documents from 17 countries (Armenia, Bangladesh, Bolivia, Cambodia, El Salvador, Ghana, Honduras, India, Indonesia, Kenya, Mexico, Nepal, Pakistan, Peru, Tanzania, Turkey and Uganda). Following allocation of documents or sub-sections by method, the review included 33 quantitative studies, 46 qualitative studies, and four economic studies. Poor quality studies were eliminated during the assessment for methodological quality but even among those that met inclusion criteria the designs and detail in reporting were often not optimal. This was particularly the case in those evaluation studies that employed diverse mixed methods and did not distinguish sources of information sufficiently clearly. We did not include reported findings for which no evidence was presented.

The review included 13 studies of maternal health components within CCTs, 31 studies of schemes that provide short-term payments to offset costs of accessing maternity services, and 22 studies of vouchers for maternity services (Table 2). Five studies of vouchers for merit goods that promote maternal health were included. Only one study of unconditional cash transfers met the inclusion criteria. Key findings on effectiveness, feasibility, appropriateness and meaningfulness are presented below [18].

**Table 2:** Modes of DSF

	**Mechanism for promoting maternal health**	**Administrative structure used**	**Distribution**	**Programmes and studies included in the systematic review**	**Monetary benefit or equivalent to recipient (maternal health component only) - expressed in US dollars and as a proportion of gross domestic product per capita per month. Illustration sourced from the most recently published study.**
**Unconditional cash transfers**	Alleviates deleterious effects of poverty on health during period of pregnancy	Government health system	Directly into a bank account	Dr Muthulakshmi Reddy Memorial Assistance Scheme (India) [14]	USD 68 (58% of GDP per capita per month) paid twice during pregnancy [14]
**Conditional cash transfers**	Conditionalities improve utilisation of specified maternity services	Social welfare system	Directly into a bank account, community distribution or sent to beneficiaries	*Oportunidades* (Mexico – previously *PROGRESA*) [11,25-32]	Not stated
				*Comunidades Solidarias Rurales* (El Salvador – previously *Red Solidaria*) [28,33]	USD 15 (5%) every month [33]
				*Juntos* (Peru) [12]	USD 75 (15%) every two months [12]
				*Bono Juana Azurduy* (Bolivia) [12]	USD 250 (129%) over 33 months [12]
				*Programa de Asignacion Familiar* (Honduras) [13]	USD 3 (3%) every month [13]
				*Social Risk Mitigation Project* (Turkey) [28]	Not stated
				*Program Keluarga Harapan *(Indonesia) [34]	USD 30 (10%) every four months [34]
**Short-term payments to offset costs of access**	Alleviates deleterious effects of poverty on access to maternity services	Government health system	Retrospective payments at health facilities	*Janani Suraksha Yojana* (India) [9,35-60]	USD 13 – 31 (11-26%), depending on location [40]
				*Safe Delivery Incentive Programme* (Nepal) [10,61,62]	USD 7 – 24 (12-41%), depending on location [10]
**Vouchers for maternity services**	Removes/reduces cost of specified maternity services at point of use	Parallel voucher management agency	Community-based distribution (if vouchers are used)	*Vouchers for Health* (Kenya) [6,63-65]	Four ANC, delivery and two PNC (voucher cost USD 2.50 - 3%) [64]
				*HealthyBaby vouchers* (Uganda) [6,66]	Four ANC, delivery and two PNC (voucher cost USD 1.20 - 3%) [66]
				*Makerere University Voucher Scheme* (Uganda) [67]	Three ANC, delivery, two PNC and transport costs (voucher provided free) [67]
				*Obstetric Care State Certificate Program* (Armenia) [68]	Aimed to eliminate informal payments [68]
				*Pilot voucher scheme* (Pakistan) [69,70]	Three ANC, delivery and one PNC (voucher cost USD 1.20 - 1%) [69]
				*Sambhav vouchers* (India) [71]	Three ANC, delivery and two PNC (voucher provided free) [71]
				*Chiranjeevi scheme* (India) [72,73]	One ANC, delivery, transport and food (no voucher)
				*MAMTA scheme* (India) [74]	Three ANC, delivery and one PNC (voucher provided free) [73]
				*Pilot voucher scheme* (Bangladesh) [75]	Three ANC, delivery, one PNC and transport costs (voucher provided free) [75]
				*Maternal Health Voucher Scheme* (Bangladesh) [8,76-80]	Three ANC, delivery, one PNC and transport costs (voucher provided free) [80]
				*Pilot voucher scheme* (Cambodia) [81]	Three ANC, delivery, one PNC and transport costs (voucher provided free) [81]
**Vouchers for merit goods**	Removes/reduces cost of merit good at point of use	Government health facilities	Distribution at health facilities	*Discount voucher scheme* (Tanzania) [82,83]	USD 0.50 (2%) discount on an insecticide-treated net costing USD 3.50 [83]
				*Tanzania National Voucher Scheme* (Tanzania) [7,84,85]	USD 2.70 (6%) discount on an insecticide-treated net costing USD 3.65 [7]
				*Volta voucher scheme* (Ghana) [86]	USD 4.20 (5%) discount on an insecticide-treated net [86]

Notes. USD refers to US dollars, ANC to antenatal care, PNC to postnatal care. Some DSF programmes fit into more than one mode, such as the Social Risk Mitigation Programme in Turkey and the Maternal Health Voucher Scheme in Bangladesh which both include short-term payments to offset costs of access for maternity services. Other programmes included supply-side components that were not reviewed here, for example removal of user fees in Nepal. Conditions were added to the Dr Muthulakshmi Reddy Memorial Assistance Scheme in 2012. Where studies did not provide a currency conversion into US Dollars, the conversion was made using historical rate tables produced by XE based on the month and year in which the article was published [87]. Value as a proportion of gross domestic product per capita per month was calculated using World Bank data from the year in which the article was published [22].

### Effectiveness of DSF to promote maternal, perinatal, neonatal and infant health outcomes

Effectiveness is described as 'the extent to which the intervention, when used appropriately, achieves the intended effect’ [18]. There was evidence of increased uptake of institutional delivery or skilled attendance at birth (Table 3), and antenatal and postnatal care (Additional file 1: Table S1A-C). Vouchers subsidising purchase of insecticide-treated bed nets increased ownership and use during pregnancy [7].

**Table 3:** Impact of DSF on skilled attendance at birth

	**Study**	**Study data**	**Effect**	**95% confidence interval, s.e. or p-value**
**Conditional cash transfers**				
	Hernandez Prado et al. [27]	2003	No effect in early intervention rural areas	p > 0.1
			20.1% increase in late intervention rural areas	p < 0.05
			10.9-11.3% relative decrease in urban areas	p < 0.05
	Urquieta et al. [32]	1998, 2000	No effect	p > 0.1
	Sosa-Rubai et al. [31]	2007	OR: 2.4 in early intervention areas	s.e.: 0.9
			OR: 3.3 in late intervention areas	s.e.: 1.4
	De Brauw and Peterman [33]	2008	12.3-17.8 percentage point increase	s.e.: 5.4-9.9
**Payments to offset costs of access**				
	Powell-Jackson et al. [61]	2001-2007	2.3 percentage point increase from baseline	p < 0.01
	Powell-Jackson and Hanson [10]	2008	16.6% increase compared to controls	CI: 4.1, 29.1
	Lim et al. [9]	2002-2004, 2007-2009	36.2-39.3 increased probability among recipients	CI: 33.7, 45.0
	Santhya et al. [45]	2009, 2010	Mean difference: 100% higher among recipients	p < 0.001
			Mean difference: 78.2% rise among recipients with past births and no increase among non-recipients	p < 0.001
	Powell-Jackson et al. [35]	2002-2004, 2007-2009	8.1 percentage point increase from baseline	s.e.: 1.8
**Vouchers for maternity services**				
	Rob et al. [75]	2007, 2008	16.1 percentage point increase from baseline	p < 0.01
	Ahmed and Khan [79]	2008	OR: 3.6 among recipients	s.e.: 0.1
	Hatt et al. [8]	2009	46.2 percentage points higher in intervention areas	p < 0.001
	Nguyen et al. [80]	2009	46.4% more likely in intervention areas	s.e.: 4.3
	Obare et al. [65]	2010	OR: 2.0 in early intervention areas	CI: 1.4, 2.8
			OR: 0.9 in late intervention areas	CI: 0.6, 1.5
	Bellows et al. [64]	2006, 2009	OR: 1.2 in intervention areas	CI: 1.0, 1.4
			OR: 12.9 among recipients	CI: 8.9, 19.3

Notes. Effect is presented as odds ratio (OR), mean difference compared to controls or percentage increase from baseline. Confidence intervals (CI) are shown if they have been provided in the study, otherwise standard errors (s.e.) and p-values are shown. No quantitative studies on unconditional cash transfers were included in the systematic review. No quantitative studies on vouchers for merit goods considered impact on skilled attendance at birth.

Evidence of effect of DSF on mortality and morbidity outcomes was sparse (Table 4). Few studies were designed to detect such effects. Two included studies investigated maternal mortality. The first, of the Mexican Oportunidades CCT programme, showed an overall 11% reduction in maternal mortality during 1995 – 2002 [25]. The second, evaluating the Bangladesh Maternal Health Voucher Scheme [8], showed no impact on maternal mortality, possibly because of small sample size. There were conflicting results on infant [25,26] and neonatal mortality [8,9,26,35,61] in part due to differing definitions and methods. No studies considered impact on maternal morbidity.

**Table 4:** Effect of DSF modes on mortality and morbidity

	**Study**	**Study data**	**Effect**	**95% confidence interval, s.e. or p-value**
***Effect on maternal mortality***				
**Conditional cash transfers**				
	Hernandez Prado et al. [25]	1995-2002	11% decrease (relative risk 0.89) compared to control areas	CI: 0.82, 0.95
**Payments to offset costs of access**				
	No studies			
**Vouchers for maternity services**				
	Hatt et al. [8]	2009	No effect compared to control areas	p = 0.42
***Effect on maternal morbidity***				
**Conditional cash transfers**				
	No studies			
**Payments to offset costs of access**				
	No studies			
**Vouchers for maternity services**				
	No studies			
***Effect on perinatal, neonatal and infant mortality***				
**Conditional cash transfers**				
	Barham et al. [26]	1992-2001	No effect on neonatal mortality	s.e.: 0.5
			17% reduction in infant mortality	p < 0.01
	Hernandez Prado et al. [25]	1995-2002	2% reduction in infant mortality	p < 0.05
**Payments to offset costs of access**				
	Lim et al. [9]	2002-2004, 2007-2009	2.3-2.4 fewer neonatal deaths per 1,000 live births	CI: 0.7, 4.1
			6.2 fewer neonatal deaths per 1,000 live births	CI: -8.1, 20.4
	Powell-Jackson et al. [35]	2002-2004, 2007-2009	No effect on neonatal mortality	p > 0.1
	Powell-Jackson et al. [61]	2001-2007	No effect on neonatal mortality	p > 0.05
**Vouchers for maternity services**				
	Hatt et al. [8]	2009	1 percentage point lower in intervention areas (stillbirths)	p < 0.001
			No effect on neonatal deaths compared to control areas	p = 0.15
***Effect on perinatal, neonatal and infant morbidity***				
**Conditional cash transfers**				
	Barber and Gertler [30]	2003	Increased average birth weight	p = 0.02
			4.6 percentage point reduction in incidence of low birth-weight	p = 0.05
	Hernandez Prado et al. [27]	2003	No effect on incidence of low birth-weight	p > 0.1
**Payments to offset costs of access**				
	No studies			
**Vouchers for maternity services**				
	No studies			

Notes. Effect is presented as odds ratio (OR), mean difference compared to controls or percentage increase from baseline. Confidence intervals (CI) are shown if they have been provided in the study, otherwise standard errors (s.e.) and p-values are shown. No quantitative studies on unconditional cash transfers were included in the systematic review. No quantitative studies on vouchers for merit goods considered impact on maternal, infant or neonatal morbidity or mortality.

### Feasibility of DSF interventions for maternal health

Feasibility is described as 'the extent to which the intervention is practical and practicable in a specific context’ [18]. Key aspects included affordability of costs to the healthcare system, availability of supporting infrastructure and the intervening effects of other demand-side barriers to access. Although designs of interventions and contexts varied, there were some general findings.

#### ***Affordability to the healthcare system***

Costs across countries and schemes were hard to compare. Overall programme costs were provided by a small number of evaluations of voucher schemes [8,71,74,84]. Costs of vouchers to subsidise the purchase of insecticide-treated bed nets were low [84], and low incremental costs were generated for vouchers for maternity services [8,71]. Short-term payments to offset costs of access have been criticised because of their relatively high expense [10]. A lack of clear benchmarks prevented assessment of whether DSF schemes could be considered low-cost interventions, or of the relative effect of similar additional spend in the supply side. In schemes involving private providers there was no evidence of competition contributing to lower costs. Accredited providers in maternal healthcare voucher schemes tended to be reimbursed at standard rates and did not compete on price [6,8,72,74]. No evidence was found on the maternal health component of CCTs.

Qualitative findings indicated the importance of high-level political and financial support to sustainability across DSF modes, with risks to schemes when regime change occurred. Long-serving CCT schemes in Latin America had relied on support of successive governments [88]. Many voucher schemes were externally funded [6,8,71], raising questions about long-term sustainability.

#### ***Availability of supporting infrastructure***

Studies of the early phases of schemes [6,62,66,76,82,83] indicated a range of supply-side and other preconditions for practicability. These included policy champions, planning using local data to inform appropriate selection criteria, incentives for providers [8,68,74] and coordination with existing interventions. Some evaluations emphasised the need for greater investment in monitoring of schemes and improved integration with the Health Management Information System [8,77]. Barriers to effective targeting included leakage of programme benefits to non-poor women [8,62] and official or unofficial requirements that restricted access for poor and socially excluded women [36-40,62,63,74,83,86,89]. Schemes often suffered from extensive bureaucratic procedures, impeding distribution of cash benefits [14,36,37,39,41-44,62], or inefficiencies in voucher distribution and provider reimbursement [8,66,74,76-78]. Some studies pointed towards utilisation of existing systems for governance and supply to encourage ownership and prevent duplication of structures [8,63,78]. Others suggested harnessing pre-existing experience in claims processing and use of a voucher management agency with clearly defined roles [6,66].

Where the motive of the DSF intervention is to increase healthcare utilisation, this is predicated on the assumption that services are of sufficient quality to improve, and not harm, maternal and perinatal health. However, there was limited evidence of the effect of DSF schemes on quality of maternity care. Only four studies attempted to quantify these effects, with inconsistent findings [8,27,45,46]. Moreover, qualitative data from a range of settings suggested that where short-term cash payments [37,89] and vouchers for maternity services [8,66,74,76] generated increased demand this placed considerable additional strain on existing staff and resources. Evaluations of DSF schemes in India highlighted the need for coordinated improvement of referral systems for complicated cases to facilitate timely and affordable transfer to higher level facilities, and reduce unnecessary transfers [38,41,43,44,47,74].

#### ***Intervening effects of other demand-side barriers to access to benefits of the schemes***

Qualitative studies showed that geographical, financial and social barriers prevented or delayed access to care via DSF schemes. Geographical remoteness and poor transport links [8,34,38-40,42,46,48-52,63,66],[81] are not resolved by DSF schemes. Additional healthcare costs not covered by a scheme, and fear of these costs, can still be prohibitive for the poor [39,53,74]. For example, some cited difficulties finding money for transport to the facility [28,42,63,75,76], transport costs for onward referrals [63,89], co-payments for vouchers [82], and medicines, tests and complex care not covered by voucher or free service arrangements [37,38,74,75,77]. Knowledge of schemes and entitlements can be increased by scheme promotion and voucher marketing locally [6,39,43,53-55,62,63,74,82],[83]. Many studies argued for community-based agents, either to encourage women to visit health facilities and claim cash payments as in Progam Keluarga Harapan in Indonesia [34] and Janani Suraksha Yojana in India [39-41,43-47,50-57] or to distribute vouchers for maternity services [6,8,74]. However, social barriers such as women’s household responsibilities can still delay uptake or cause early self-discharge from hospital, and need to be addressed with wider social interventions [12,56,72,81].

Poor behaviour of healthcare staff actively deterred women from using DSF schemes or resulted in negative experiences [8,12,38,40,45,48,50,56],[63,81,85,89]. Of 12 studies that referred to poor behaviour among health care staff, half were of state level implementation of India’s Janani Suraksha Yojana programme, but the problem appears to be widespread: one study related to CCTs in Bolivia and Peru [12], one to vouchers for insecticide-treated nets in Tanzania [85], and the remaining three studies were on vouchers for maternity services in Bangladesh, Cambodia and Kenya [8,63,81]. Where schemes had successfully increased demand for services, poor attitudes were compounded by extended waiting times and shortages of supplies [8,12,40,45,48,53,54,75],[89]. Of the nine studies that supported this finding, six related to India’s Janani Suraksha Yojana [40,45,48,53,54,89], one related to CCTs in Bolivia and Peru [12] and two were on Bangladesh’s Maternal Health Voucher Scheme [8,75]. Corrupt practices also diluted benefits of DSF schemes and reduced the trust of potential users. Evidence concerning short-term payments to offset costs of access in India [37-39,42,45,47,49,50,53,74], and Nepal [62], and vouchers for maternity services in Cambodia [81], Kenya [63] and Armenia [68] indicated that some healthcare providers kept cash payments meant for women, demanded informal payments, or falsified claims to programme management agencies. There was also evidence of voucher leakage in Tanzania National Voucher Scheme, with up to half of the vouchers being misused by issuing clinics [83].

### Appropriateness of DSF interventions for maternal health

Appropriateness is described as the 'extent to which the intervention fits with or is apt in a situation’ [18]. Evidence was identified concerning three aspects of appropriateness: DSF schemes’ fit to the situations of the intended beneficiaries, their fit to providers of services, and their fit to population health objectives and social justice.

#### ***Intended beneficiaries***

Several studies indicated that inappropriate eligibility criteria or distribution channels restricted access for poor, rural or socially excluded women in Bangladesh [8,76], India [38] and Nepal [62], and the partial nature of the voucher subsidy for insecticide-treated bed nets in Tanzania limited purchase by poor women [82]. Some studies highlighted the importance of concurrent information strategies to raise family awareness of the need for maternal healthcare [28,75,82,86].

#### ***Providers of services***

Scheme designs need to take the effects on healthcare providers into account. Due consideration needs to be given to prevention of perverse incentives [72,74] and to whether an increased workload could be balanced with other rewards [42,63,67,72,74]. Weaknesses in monitoring allowed distortions to take place, such as over-reporting of activity [38,45,58,62,68,83]. In the Chiranjeevi scheme in Gujarat, providers are reimbursed with a flat rate regardless of complications and this led to a reluctance to deal with complicated cases ('cream-skimming’) [72]. Schemes involving the opt-in of private providers were vulnerable to attrition if they were less rewarding that initially anticipated [63,67,74].

#### ***Social justice***

Assessment of appropriateness of the DSF schemes is more complex when viewed from a social justice perspective. Some DSF schemes simply aimed to increase coverage of facility delivery, in response to Millennium Development Goal indicators [9,10,73,76]. Some, such as the Obstetric Care State Certificate Program in Armenia, were reported to offer women a greater sense of dignity and of their entitlement to care [68]. However, qualitative findings indicated that eligibility criteria employed in several of the DSF schemes were problematic. For example, in order to reward family planning some schemes confined eligibility to women who had used contraception for birth spacing, effectively restricting women’s right to reproductive choice [8,12]. Others confined eligibility to women with a small number of live children thereby excluding the poorest [62]. Schemes that incorporated existing targeting mechanisms such as India’s 'below poverty line’ card were practical but required safeguards to ensure that undocumented women were not unfairly excluded [14,38].

Most early DSF schemes had insufficient reach to protect poor households from catastrophic costs of obstetric complications. For example in Nepal’s Makwanpur district, despite the Safe Delivery Incentive Programme, caesarean section costs accounted for 19% of total annual household consumption [61]. Among households where a woman had caesarean section delivery, the incidence of catastrophic expenditure (>10% of total annual household consumption) across wealth quintiles was 72% [61]. Across DSF options, voucher schemes were the most easily adapted to cover common birth complications [69,70].

### Meaningfulness of DSF interventions for maternal health

Meaningfulness is described as the 'extent to which the intervention is positively experienced by the user’ [18]. Studies reported evidence on targeting and stigma, respect, and contribution to improving women’s status.

#### ***Targeting, stigma and respectful treatment***

Some study findings suggested that targeted schemes could be implemented without stigmatising the user. Five studies reported women as saying that their needs were given greater consideration by family members and health facility staff as a result of DSF schemes [8,14,34,45,68]. These studies related to an unconditional cash transfer programme in the Indian state of Tamil Nadu [14], a CCT programme in Indonesia [34], India’s Janani Suraksha Yojana in the state of Rajasthan [45] and vouchers for maternity services in Armenia and Bangladesh. However, there were also many accounts of disrespectful treatment by healthcare providers when women used DSF schemes to access services [8,12,38,40,45,48,50,56],[63,81,85,89]. More than half of these 12 studies were of state level implementation of India’s Janani Suraksha Yojana [38,40,45,48,50,56,89], but the issue was also identified in studies relating to the Tanzania National Voucher Scheme for insecticide-treated nets [85], CCTs in Bolivia and Peru [12] and vouchers for maternity services in Bangladesh, Cambodia and Kenya [8,63,81]. Where private facilities were perceived to be of better quality than those in the public sector, users valued the access that some voucher schemes gave to such a facility [74].

#### ***Neglected opportunities to improve women’s social position***

Some studies noted lost opportunities for DSF schemes to contribute to wider conditions necessary for maternal health, such as strengthening women’s status. These cited cash intended for women being given to family members [41,89]; increased work imposed on women by conditionalities (CCT schemes) [12]; failures to involve women’s organisations in design of DSF schemes [12,44]; and failures to take on issues such as gender-based violence within obligatory health education sessions (CCT schemes) [12].

## Discussion

The primary review objective, to assess effects of DSF interventions on maternal healthcare utilisation and outcomes in low- and middle-income countries, was achieved in part for CCTs, for cash payments to offset costs of accessing maternal healthcare, and for vouchers for maternity services. This systematic review found little research evidence on unconditional cash transfers, and very limited evidence on vouchers for merit goods. Evidence on effect on maternal and infant mortality and morbidity outcomes was insufficient with most studies to date too small or the follow-up period too short to assess impacts on health outcomes. There is also a lack of evidence on cost-effectiveness.

We sought to move beyond the constraints of previous systematic reviews for modes of DSF and to learn from existing experiences with implementation in a wide range of settings by using a comprehensive set of review questions and by adopting a more inclusive approach to studies. Some of the included studies and evaluations therefore did not have optimal research designs or lacked comprehensive detail on elements of their design and fieldwork processes. An additional limitation is that search terms were restricted to English and conducted in English language databases, although we are confident that we captured a range of experiences as more than one-fifth of the studies included in the review examined schemes in non-English speaking countries. Restriction of searches to publications from 1990 onwards could have excluded relevant earlier studies. The focused India searches revealed useful additional evidence on experiences of large and smaller-scale schemes but may have given that country greater emphasis than others as a result. One of the biggest challenges has been how to give due recognition to the variety of contexts and interventions represented across the studies in 17 countries.

The evidence we have identified and reviewed does indicate that a range of different DSF schemes have the capacity to increase utilisation of maternal healthcare in the index pregnancy or to increase uptake of related merit goods. This complex review has also brought together evidence on implementation processes and challenges from 17 countries. Analysis using the different JBI facets produced useful pointers for policy. Targeting and eligibility criteria, monitoring, respectful treatment of beneficiaries, suitable incentives for providers, and the need for investment in quality of care and affordable referral systems were highlighted as areas needing detailed and context-specific attention. Much evidence came from early evaluations and some schemes have since advanced in sophistication. The Nepal Safe Delivery Incentive Programme [10] incorporated incentive payments to health workers and cash payments to women intended to offset other costs. Indira Gandhi Matritva Sahayog Yojana in India [90] included additional maternal health components such as a cash incentive for nutrition during pregnancy. Greater complexity in turn introduces new challenges for effective administration.

Outside of India’s Janani Suraksha Yojana many of the DSF schemes that were specifically focused on maternal health have been donor-assisted or sponsored. The insufficient evidence on cost-effectiveness and preconditions for sustainability and scale-up of DSF schemes means that the findings on positive effects need to be treated with some caution. DSF programmes undergoing scale-up in Kenya, Uganda and Bangladesh may provide potential sites for further evaluations. Other research gaps include:

• Short and longer-term impact of DSF on maternal and infant mortality and morbidity;

• The effects of unconditional social transfers and vouchers for food on maternal health and other outcomes;

• The effects of vouchers for food on maternal health and other outcomes;

• The quality and safety of services provided to beneficiaries of DSF schemes, including maternity care experiences of the user, and how these can be optimised;

• The specific effects of DSF schemes for poor, rural and socially excluded women;

• Mechanisms to involve women’s organisations and other user representatives in design and implementation and monitoring of DSF schemes;

• Any effect of DSF interventions on competition in markets for the provision of health services;

• The optimal and most practical administration systems for DSF programmes at different stages of scale up, to avoid duplication and undue expense while maintaining efficiency and transparency, and

• The cost-effectiveness of DSF interventions, comparing with similar investment in supply-side financing mechanisms.

Research analyses of policy processes are also still required. India’s Janani Suraksha Yojana exemplifies centrally-driven scale-up of a cash incentive scheme integrated within the public healthcare sector, but allowing state-level variations in implementation. Mexico’s PROGRESA/Oportunidades offers a quite different scenario as it does not have maternal health as its primary focus but rather as a sub-component of a national scheme to address child poverty and disadvantage, but it can provide general lessons on how to embed a monitoring and rigorous evaluation structure within a large scale long-term programme. Finally, it is important to point out that the paucity of comparative studies [13] limits our ability to assess DSF schemes against alternative routes to achieving the same objectives, for example by investment in the supply-side of healthcare or by removal of user fees.

## Conclusions

The findings from the synthesis of evidence on short-term cash payments and vouchers for maternity services, and on CCTs with maternal health conditionalities, suggest seven key messages for future directions for schemes employing DSF for maternal health.

First, there is good evidence that DSF modes such as cash payments, vouchers and CCTs can help to increase the use of priority maternity services including births in healthcare facilities. However, other social, geographical and financial barriers to access limit the impact of DSF, and concurrent initiatives are required to reduce such barriers and ensure high quality care is provided.

Second, there is some evidence that attempts to utilise DSF to simultaneously address complex and multiple policy objectives are unlikely to be achieved, and may be counter-productive. For example, schemes such those in Bangladesh, India and Nepal that restricted the benefits to sub-groups such as women with fewer than two previous children in order to fit with national government policies on family size, act to exclude the poorest families that have more children.

Third, targeting should be as simple as possible, using existing systems – for example India’s Below Poverty Line card - for identifying beneficiaries. Additional efforts should be made to ensure identification and inclusion of those on the social margins who do not, for one reason or another, possess necessary official documentation or who are otherwise systematically excluded by the targeting system.

Fourth, there is little evidence that DSF mechanisms alone can be used to improve quality of care in maternal health provision. Demand-side financing measures increase demand for services in most situations and this places added strain on existing scarce resources. While vouchers for maternity services offer opportunities to be linked directly to output-based payments to providers that can be used for the strengthening of supply-side quality of care, other forms of DSF require concurrent investment in the supply-side to maintain and improve quality of basic care. In many schemes insufficient consideration was given to the needs of women who require complicated care during childbirth. Supply-side investment is required to develop referral systems for complicated cases, for expensive support services such as blood banking, as well as for bed capacity.

Fifth, qualitative findings suggest that success in initiating, sustaining, and scaling-up schemes is highly dependent on a good understanding of what works in that context. Frequently, scheme feasibility was reduced by extensive bureaucratic procedures that caused delays or by inefficiencies in voucher distribution and provider reimbursement systems. There is also evidence that, as with other health programmes in many settings, poorly designed systems permit or encourage corrupt practices. These included informal fees demanded from patients in return for services ostensibly covered by schemes, deductions taken from cash payments to beneficiaries, unjustifiable referral-on of unwanted patients, or over-reporting of numbers of cases received.

Pilot phases often contract out financing activities to a non-government body with expertise in identifying beneficiaries and disbursing funds. Little comparative data currently exists, but scaling-up requires due attention to the relative costs and benefits of continuing to administer the enlarged scheme in this way or of incorporation of the scheme into the government system.

Sixth, substantial preparation is required to ensure that health facilities can adequately administer schemes. Evidence also suggests that many of the logistical problems in administering DSF arose because potential beneficiaries did not know about schemes or did not understand how they functioned. Corruption also undermined the ability of schemes to reduce recipients’ out-of-pocket expenditure. This, and lack of transparency, reduces user trust. Community-level behaviour change communication campaigns to inform households may be needed in order to improve knowledge, and to maximise uptake of benefits by those in most need.

Seventh, insufficient attention has been paid to respect for beneficiaries and to gender issues in most DSF schemes. While some women did report receiving good care at facilities, qualitative findings across various studies indicated that disrespectful treatment from healthcare staff was a common experience for poor women seeking care under DSF schemes, and that such behaviour went largely unchallenged.

Some women, in some studies, felt that they were treated with more consideration by their families, or that their sense of entitlement was enhanced as a result of their status as beneficiaries of DSF schemes. However, several studies highlighted missed opportunities for schemes to address gender inequalities, such as failure to actively involve local women’s organisations in programme design and implementation, or failure to take on gender-based violence within the health education sessions linked to CCTs. Attention needs to be paid to how schemes act to reinforce or reduce existing social inequalities.

## Abbreviations

CCT: Conditional cash transfer; DSF: Demand-side financing; JBI: Joanna Briggs Institute.

## Competing interests

No known competing interests for SFM, BH, RB and DB. TE was involved in some of the early design work of DSF schemes in Nepal and also in Bangladesh, and more recently was involved in commissioning and commenting on the evaluation of both schemes. No other conflicts of interest.

## Authors’ contributions

SFM and DB designed the study. BH designed and conducted the systematic searches and constructed the datasets. SFM, BH and DB conducted the literature search, methodological appraisals, analyses and synthesis. RB led a systematic search of Indian grey literature and contributed to analysis. TE contributed towards the study design, methodological appraisal and synthesis. All authors contributed to the writing of the manuscript. All authors read and approved the final manuscript.

## Pre-publication history

The pre-publication history for this paper can be accessed here:

http://www.biomedcentral.com/1471-2393/14/30/prepub

## Supplementary Material

Additional file 1 **Table A.** Effect of DSF modes on antenatal care. Table B. Effect of DSF modes on postnatal care. Table C. Details of included studies. Click here for file
