# Supplementary material for: Effects of demand-side financing on utilisation, experiences and outcomes of maternity care in low- and middle-income countries: a systematic review
Source: BMC Pregnancy Childbirth. 2014 Jan 17;14:30. doi: 10.1186/1471-2393-14-30 (PMC3897964; doi:10.1186/1471-2393-14-30)
Supplement: Additional file 1 — Table A. Effect of DSF modes on antenatal care. Table B. Effect of DSF modes on postnatal care. Table C. Details of included studies. [file 1471-2393-14-30-S1.docx]

**Supplementary tables**

| **Supplementary Table A. Effect of DSF modes on antenatal care** | | | | | |
| --- | --- | --- | --- | --- | --- |
|  | **Study** | **Study data** | **Effect** | **95% confidence interval, s.e. or p-value** | |
| **Conditional cash transfers** | | | | | |
|  | ***Any antenatal care*** | | | | |
|  | Hernandez Prado et al. [[54](#_ENREF_54)] | 2003 | No effect in early intervention areas relative to control areas  6.7% increase in late areas relative to control areas  No effect in urban areas relative to control areas | | p>0.05  p<0.05  p>0.1 |
|  | Barber and Gertler [[82](#_ENREF_82)] | 2003 | No effect | | p=0.12 |
|  | ***Mean number of visits*** | | | | |
|  | Hernandez Prado et al. [[54](#_ENREF_54)] | 2003 | 11.9% greater increase in early rural areas relative to control areas  No effect in late rural areas relative to control areas  No effect in urban areas relative to control areas | | p<0.05  p>0.1  p>0.1 |
|  | Barber and Gertler [[82](#_ENREF_82)] | 2003 | No effect | | p=0.42 |
|  | Sosa-Rubâi et al. [[83](#_ENREF_83)] | 2007 | OR: 0.98 in early intervention areas  OR: 1.02 in late intervention areas | | s.e.: 0.04  s.e.: 0.05 |
|  | ***Five or more visits*** | | | | |
|  | Hernandez Prado et al. [[54](#_ENREF_54)] | 2003 | 24.6% increase in early rural areas relative to control areas  27.9% increase in late rural areas relative to control areas | | p<0.05  p<0.01 |
|  | Morris et al. [[13](#_ENREF_13)] | 2000, 2002 | 18.7% increase in intervention areas | | CI: 7.4, 30.0 |
|  | Barber and Gertler [[82](#_ENREF_82)] | 2003 | No effect | | p=0.35 |
|  | De Brauw and Peterman [[85](#_ENREF_85)] | 2008 | No effect | | p>0.1 |
| **Payments to offset costs of access** | | | | | |
|  | ***Three or more visits*** | | | | |
|  | Lim et al. [[9](#_ENREF_9)] | 2002-2004, 2007-2009 | 10.7-11.1% higher odds among recipients | | p<0.05 |
|  | Santhya et al. [[55](#_ENREF_55)] | 2009, 2010 | OR: 2.2 among recipients  7% increase using difference-in-difference analyses | | CI: 1.9, 2.6  CI: 0.02, 0.14 |
|  | Powell-Jackson et al. [[27](#_ENREF_27)] | 2002-2004, 2007-2009 | 8.8% increase | | p<0.1 |
| **Vouchers for maternal health services** | | | | | |
|  | ***Any antenatal care*** | | | | |
|  | Bellows et al. [[88](#_ENREF_88)] | 2006, 2009 | OR: 0.7 in intervention areas  OR: 16.5 among recipients | | CI: 0.5, 1.0  CI: 4.0, 68.1 |
|  | ***Three or more visits*** | | | | |
|  | Agha [[78](#_ENREF_78)] | 2010 | OR: 5.0 among recipients | | p<0.001 |
|  | Agha [[77](#_ENREF_77)] | 2009, 2010 | OR: 1.6 in intervention areas | | p<0.01 |
|  | Rob et al. [[68](#_ENREF_68)] | 2007, 2008 | 33.2 percentage point increase from baseline | | p<0.01 |
|  | Hatt et al. [[8](#_ENREF_8)] | 2009 | 25.3 percentage point greater increase than in control areas | | p<0.001 |
|  | Nguyen et al. [[90](#_ENREF_90)] | 2009 | 24.1% more likely than in control areas | | s.e.: 7.6 |
|  | Ahmed and Khan [[57](#_ENREF_57)] | 2008 | OR: 1.9 among recipients | | s.e.: 0.13 |
|  | ***Four or more visits*** | | | | |
|  | Obare et al. [[89](#_ENREF_89)] | 2010 | OR: 1.1 in early intervention areas  OR: 0.9 in late intervention areas | | CI: 0.8, 1.6  CI: 0.7, 1.4 |
|  | Bellows et al. [[88](#_ENREF_88)] | 2006, 2009 | OR: 0.7 in intervention areas  OR: 1.9 among recipients | | CI: 0.6, 0.8  CI: 1.6, 2.4 |
|  | Reproductive Health Vouchers Evaluation Team [[37](#_ENREF_37)] | 2008, 2010-2011 | 10 percentage point increase from baseline in intervention areas | | p<0.01 |

Notes. Effect is presented as odds ratio (OR), mean difference compared to controls or percentage increase from baseline. Confidence intervals (CI) are shown if they have been provided in the study, otherwise standard errors (s.e.) and p-values are shown. No quantitative studies on unconditional cash transfers were included in the systematic review. No quantitative studies on vouchers for merit goods considered impact on antenatal care.

| **Supplementary Table B. Effect of DSF modes on postnatal care** | | | | | |
| --- | --- | --- | --- | --- | --- |
|  | **Study** | **Study data** | **Effect** | **95% confidence interval s.e. or p-value** | |
| **Conditional cash transfers** | | | | | |
|  | Morris et al. [[13](#_ENREF_13)] | 2000, 2002 | 5.6% reduction in intervention areas | | CI: -4.5, 15.7 |
|  | De Brauw et al. [[85](#_ENREF_85)] | 2008 | No effect | | p>0.1 |
| **Payments to offset costs of access** | | | | | |
|  | Santhya et al. [[55](#_ENREF_55)] | 2009, 2010 | OR: 8.2 among recipients  39% increase among women with previous births | | CI: 7.0, 9.7  CI: 32, 46 |
| **Vouchers for maternal health services** | | | | | |
|  | Agha [[78](#_ENREF_78)] | 2010 | OR: 5.8 among recipients | | p<0.001 |
|  | Agha [[77](#_ENREF_77)] | 2009, 2010 | OR: 1.3 | | p>0.05 |
|  | Rob et al. [[68](#_ENREF_68)] | 2007, 2008 | 14.9 percentage point increase from baseline | | p<0.01 |
|  | Hatt et al. [[8](#_ENREF_8)] | 2009 | 19.6 percentage point greater increase than in control areas | | p<0.001 |
|  | Nguyen et al. [[90](#_ENREF_90)] | 2009 | 19.1% more likely than in control areas | | s.e.: 3.5 |
|  | Ahmed and Khan [[57](#_ENREF_57)] | 2008 | OR: 2.8 among recipients | | s.e.: 0.14 |
|  | Obare et al. [[89](#_ENREF_89)] | 2010 | OR: 1.3 in early intervention areas  OR: 0.8 in late intervention areas | | CI: 0.9, 1.8  CI: 0.6, 1.2 |
|  | Reproductive Health Vouchers Evaluation Team [[37](#_ENREF_37)] | 2008, 2010-2011 | 8 percentage point increase from baseline in intervention areas | | p<0.01 |

Notes. Effect is presented as odds ratio (OR), mean difference compared to controls or percentage increase from baseline. Confidence intervals (CI) are shown if they have been provided in the study, otherwise standard errors (s.e.) and p-values are shown. No quantitative studies on unconditional cash transfers were included in the systematic review. No quantitative studies on vouchers for merit goods considered impact on postnatal care.

| **Supplementary Table C. Details of included studies** | | | | | | |
| --- | --- | --- | --- | --- | --- | --- |
| **Study** | **Participants** | **Intervention** | **Context** | **Study size** | **Risk of bias** | **Relevant outcomes** |
| **Quantitative studies** | | | | | | |
| Agha, S., 2011 | Randomly selected women who had given birth in the 12 months preceding the survey | Pilot voucher scheme (vouchers for maternal health services) | Dera Ghazi Khan City (urban), Pakistan | Baseline: 681  Follow-up: 742 | High  No control areas  Effect of repeated visits by voucher distributors | 3 x ANC, ID, any PNC |
| Agha, S., 2011 | Randomly selected women who had given birth in the 12 months preceding each survey | Pilot voucher scheme (vouchers for maternal health services) | Jhang district (rural), Pakistan | Baseline: 2,018  Follow-up: 2,033 | Moderate  Purposive selection of intervention areas  Effect of repeated visits by voucher distributors | 3 x ANC, ID, any PNC |
| Ahmed, S. and M.M. Khan, 2011 | Women who had given birth within a year prior to the survey | MHVS (vouchers for maternal health services) | Sarishabari district (predominantly rural), Bangladesh | 3,600 | Low  Included measures of equity | 3 x ANC, SBA, ID, any PNC |
| Barber, S.L. and P.J. Gertler, 2008 | Poor women aged 15-49 in poor rural communities | Oportunidades (conditional cash transfers) | Rural communities, Mexico | 840 | Low  Risk of recall bias | Average BW, prevalence of low BW, any ANC |
| Barber S.L. and P.J. Gertler, 2009 | Poor women aged 15-49 in poor rural communities | Oportunidades (conditional cash transfers) | Rural communities, Mexico | 892 | Low  Quality defined as receipt of specific procedures | ANC procedures |
| Barber, S.L., 2010 | Poor women aged 15-49 in poor rural communities | Oportunidades (conditional cash transfers) | Rural communities, Mexico | 979 | Low  Rate of CS remained <15% despite increase | CS |
| Barham, T., 2011 | Poor women aged 15-49 in poor rural communities | Oportunidades (conditional cash transfers) | Rural communities, Mexico | 19,421 | Low  Possible weaknesses in reporting | Infant mortality, neonatal mortality |
| Bellows et al, 2011 | Females aged 12–54 years who had given birth during the two years preceding the surveys | Vouchers for Health (vouchers for maternal health services) | Informal settlements in Nairobi (urban), Kenya | Baseline: 1,914  Follow-up: 2,448 | Moderate  No control areas | Any ANC, 4 x ANC, SBA, ID |
| Bhat, R. et al., 2009 | Women in households selected from an intervention district | Chiranjeevi Scheme (vouchers for maternal health services) | Dahod district (mixed urban and rural), India | 656 | Moderate  No accounting for confounding factors | Any PNC |
| de Brauw et al, 2011 | Households with children under 3 years old or a pregnant woman | Comunidades Solidarias Rurales (conditional cash transfers) | Rural communities, El Salvador | Baseline: 269  Follow-up: 287 | Low  Concurrent increases in service availability | 5 x ANC, SBA, ID, any PNC |
| Hanson et al., 2009 | Women aged 15-49 | Tanzanian National Voucher Scheme (vouchers for merit goods) | 21 districts (mixed urban and rural), Tanzania | 2005: 6,199  2006: 6,260  2007: 6,198 | Low  Concurrent ITN distribution programmes | ITN ownership and use |
| Hatt et al, 2010 | Eligible women who had delivered in the six months preceding the survey | Maternal Health Voucher Scheme (vouchers for maternal health services) | Early implementation subdistricts (mainly rural), Bangladesh | 2,837 | Low  Questionable power to detect mortality | 3 x ANC, ID, CS, any PNC, QoC, personal expenditure |
| Hernandez Prado et al., 2004 | Municipalities with one or more household enrolled in Oportunidades | Oportunidades (conditional cash transfers) | Rural, semi-urban and urban areas, Mexico | 2,445 municipalities | Low  Risk of underlying confounders | Maternal mortality, infant mortality |
| Hernandez Prado et al., 2004 | All poor women aged 15-49 eligible to be incorporated into Oportunidades | Oportunidades (conditional cash transfers) | Rural, semi-urban and urban areas, Mexico | 1998-2000: 29,041  2003: 7,802 | Moderate  Wide variation of results within study | Any ANC, average ANC, 5 x ANC, SBA, ID, QoC |
| Lim et al., 2010 | Ever-married women aged 15-44 | Janani Suraksha Yojana (payments to offset costs of access) | National sample of districts (mixed urban and rural), India | 2002-2004: not provided  2007-2009: 182,869 | Low  Data collected very shortly after launch  Includes non-JSY payments | 3 x ANC, SBA, ID, Perinatal mortality, neonatal mortality |
| Morris et al., 2004 | Pregnant women and mothers of children younger than 3 years old | Programa de Asignación Familiar (conditional cash transfers) | Rural municipalities, Honduras | 11,002 households | Low  Results not verified by government data | 5 x ANC, any PNC |
| Nandan et al, 2010 | Representatives from private providers involved in the MAMTA scheme | MAMTA scheme (vouchers for maternal health services) | Delhi National Capital Territory (urban), India | 35 | Low  Descriptive statistics from 35 of the 36 providers | Satisfaction with programme |
| National Health System Resource Centre, 2011 | Women who had given birth in the 12 months preceding the survey and received benefits through JSY | Janani Suraksha Yojana (payments to offset costs of access) | Districts in 8 states, India | 2,759 | Moderate  No accounting for confounding factors | Personal expenditure |
| Nguyen et al, 2012 | Women who gave birth in the 6 months preceding the survey | Maternal Health Voucher Scheme (vouchers for maternal health services) | Early implementation subdistricts (mainly rural), Bangladesh | 2,208 | Low  No indication of voucher uptake | 3 x ANC, SBA, ID, any PNC |
| Obare et al, 2012 | Women aged 15-49 who gave birth in the 12 months preceding the survey (or was pregnant at the time of the survey) | Vouchers for Health (vouchers for maternal health services) | 6 districts (all mixed urban and rural), Kenya | 2,527 | Moderate  Leakage of vouchers to non-poor women  Respondents selected within 5 kilometres of a health facility | 4 x ANC, SBA, ID, any PNC |
| Powell-Jackson et al., 2009 | Every woman who gave birth in the study district | Safe Delivery Incentive Programme (payments to offset costs of access) | Makwanpur district (rural), Nepal | 14,799 | Low  P-values not clear | SBA, ID, neonatal mortality, personal expenditure, |
| Powell-Jackson et al., 2011 | Ever-married women aged 15-44 | Janani Suraksha Yojana (payments to offset costs of access) | 6 districts (mixed urban and rural, Nepal | 5,903 | Low  Data collected very shortly after launch | 3 x ANC, SBA, ID, neonatal mortality |
| Powell-Jackson and Hanson, 2012 | Women who had given birth during the 3 years before the survey | Safe Delivery Incentive Programme (payments to offset costs of access) | National sample of districts (mixed urban and rural), India | 2002-2004: 507,622  2007-2009: 643,944 | Low  Risk of recall bias | SBA, ID, type of provider used |
| Reproductive Health Vouchers Evaluation Team, 2012 | Women aged 15-49 years who had a pregnancy or birth during the 12 months preceding the survey and men whose partner was eligible | HealthyBaby vouchers (vouchers for maternal health services) | 6 districts, Uganda | Baseline: 2,443  Follow-up: 2,895 | Moderate  No control areas | 4 x ANC, ID, any PNC, personal expenditure |
| Rob et al., 2009 | Poor women who had given birth in the 12 months preceding each survey (endline included only recipients) | Pilot voucher scheme (vouchers for maternal health services) | Habiganj district (rural), Bangladesh | Baseline: 436  Follow-up: 414 | Moderate  No accounting for confounding factors | 3 x ANC, SBA, ID, any PNC |
| Santhya et al., 2011 | Women aged below 35 years who had given birth in the 12 months preceding the survey | Janani Suraksha Yojana (payments to offset costs of access) | Alwar and Jodhpur districts (mixed rural and urban), India | 4,770 | Moderate  Respondent selection methods unclear | 3 x ANC, SBA, ID, any PNC, QoC, personal expenditure |
| UNFPA India, 2009 | Women who had given birth in the 12 months preceding the survey | Janani Suraksha Yojana (payments to offset costs of access) | Rural areas in five states, India | 6,002 | Moderate  Simple analysis of out of pocket expenditure | Personal expenditure |
| Uttekar et al. 2007 | Recipients of JSY payments | Janani Suraksha Yojana (payments to offset costs of access) | Three districts in Himachal Pradesh, India | 237 | Moderate  Simple analysis of out of pocket expenditure | Personal expenditure |
| Uttekar et al. 2007 | Recipients of JSY payments | Janani Suraksha Yojana (payments to offset costs of access) | 3 districts in Assam, India | 240 | Moderate  Simple analysis of out of pocket expenditure | Personal expenditure |
| Uttekar et al. 2007 | Recipients of JSY payments | Janani Suraksha Yojana (payments to offset costs of access) | 3 districts in West Bengal, India | 480 | Moderate  Simple analysis of out of pocket expenditure | Personal expenditure |
| Uttekar et al. 2007 | Community health workers and recipients of JSY payments | Janani Suraksha Yojana (payments to offset costs of access) | 3 districts in Orissa, India | 178 health workers  245 recipients | High  No baseline data | QoC |
| **Economic studies** | | | | | | |
| Hatt et al, 2010 | Representatives of the Ministry of Health and Family Welfare, WHO and DFID, and programme administrators. | Maternal Health Voucher Scheme (vouchers for maternal health services) | Early implementation subdistricts (mainly rural), Bangladesh | 16 subdistricts where the quantitative study was conducted | Low  Cost to patients not included  No comparators provided | Cost of programme, cost per voucher distributed |
| IFPS Technical Assistance Project, 2012 | Health service administrators, voucher management agency, community leaders, health workers and women who received vouchers | Sambhav voucher scheme (vouchers for maternal health services) | Haridwar district in Uttarakhand, India | One district  246 interviews with participants | Low  No comparators provided | Cost per voucher used |
| Mulligan et al., 2008 | Project staff, providers and women who used the vouchers | Tanzania National Voucher Scheme (voucher for merit goods) | Tanzania | National programme | Low  Used an established framework for evaluation | Cost of programme, cost per ITN distributed |
| Nandan et al, 2010 | Hospital accredited within the programme | MAMTA scheme (vouchers for maternal health services) | Delhi National Capital Territory, India | One hospital | High  No details on hospital and representativeness | Average hospital costs per patient |
| **Qualitative studies** | | | | | | |
| Adato, M. et al., 2011 | Mexico: Focus groups with women from poor communities; interviews with health workers  El Salvador and Turkey: ethnographic work in programme communities | Oportunidades, Red Solidaria, and the Social Risk Mitigation Program | 6 states (rural areas) in Mexico  6 rural communities in El Salvador and 6 urban and rural communities in Turkey | Mexico: 23 focus groups and 16 interviews  El Salvador: 10 case study households, 96 interviews and clinic observations  Turkey: 10 case study households and 87 interviews | Low  No indication of ethical approval | Barriers |
| Ahmed S and MN Khan, 2011 | Managers, Union Committee members, service providers, voucher distributors and beneficiaries | Maternal Health Voucher Scheme (vouchers for maternal health services) | Sarishabari district (predominantly rural), Bangladesh | 13 semi-structured interviews | Low  Not clear who conducted interviews and what effect they had | Barriers, provider experiences, preconditions, scale up and sustainability |
| Arur, A. et al, 2009 | Technical support teams associated with each programme | Vouchers for Health and HealthyBaby vouchers (vouchers for maternal health services) | Kenya and Uganda | Number of semi-structure interviews not provided | Moderate  Difficult to discern findings from authors’ opinions | Barriers, preconditions |
| Chaturvedi and Randive, 2009 | Semi-structured interviews with public and private providers, recipients and non-recipients; focus groups with providers | Janani Suraksha Yojana (payments to offset costs of access) | Ahmednagar district in Maharashtra, India | 37 semi-structured interviews  2 focus groups | Moderate  No indication of researchers’ impact on findings  No indication of ethical approval | Barriers, ethical issues, social meaning, preconditions |
| Chaturvedi and Randive, 2011 | Programme managers, providers, pregnant women eligible for JSY payments | Janani Suraksha Yojana (payments to offset costs of access) | Ahmednagar district in Maharashtra, India | 37 semi-structured interviews  2 focus groups | Low  No indication of researchers’ impact on findings | Barriers, provider experiences, preconditions |
| Dasgupta, J., 2007 | Cases of women who had experienced adverse maternal health outcomes, including neonatal death, maternal death and maternal morbidities | Janani Suraksha Yojana (payments to offset costs of access) | 7 districts in Uttar Pradesh, India | 20 cases investigated | Moderate  No indication of researchers’ impact on findings  No indication of ethical approval | Social meaning |
| Devadasan et al., 2008 | Programme managers, health workers and recipients | Janani Suraksha Yojana (payments to offset costs of access) | One district in each of four states, India | 90 interviews | Moderate  Limited detail provided on methods  Purposive selection of district not explained | Barriers, provider experiences, ethical issues, preconditions |
| Febriany et al, 2011 | Service providers, program implementers, community leaders and recipients | Program Keluarga Harapan (conditional cash transfer) | 12 villages (mixed urban and rural) in 2 provinces, Indonesia | Unclear  Up to 192 interviews and 96 focus groups | Moderate  Difficult to attribute changes to DSF alone  No indication of ethical approval | Barriers, provider experiences, ethical issues, social meaning, preconditions |
| Gupta, A., 2007 | In-depth interviews with eligible women and community health workers. Focus group discussions with eligible women | Janani Suraksha Yojana (payments to offset costs of access) | Nalanda and West Champaran (rural) districts in Bihar, India | Up to 40 interviews or several focus groups | Moderate  Limited detail provided on methods  No indication of researchers’ impact on findings | Provider experiences, ethical issues, social meaning |
| Hangmi and Kuki, 2009 | Semi-structured interviews with eligible women and providers; focus group discussions with eligible women and health workers | Janani Suraksha Yojana (payments to offset costs of access) | Churachandpur (rural) district in Manipur, India | 55 interviews  Unclear how many focus groups were conducted | Moderate  Limited detail provided on methods  No indication of researchers’ impact on findings  No indication of ethical approval | Provider experiences, social meaning |
| Hatt et al, 2010 | Interviews with programme managers, voucher distributors, health workers, NGO and private sector representatives; focus groups with recipients and non-recipients | Maternal Health Voucher Scheme (vouchers for maternal health services) | 24 early implementation subdistricts, Bangladesh | 147 interview  10 focus groups | Low  No indication of researchers’ impact on findings | Barriers, provider experiences, ethical issues, social meaning, preconditions |
| Human Rights Watch, 2009 | Beneficiaries and non-beneficiaries, service providers, officials, journalists and activists | Janani Suraksha Yojana (payments to offset costs of access) | Rural areas in Uttar Pradesh, India | 95 individuals  96 group interviews | Low  Representativeness of findings unclear | Barriers, provider experiences, ethical issues, social meaning, preconditions |
| Ir et al., 2010 | Interviews and focus groups with voucher recipients | Pilot voucher scheme (vouchers for maternal health services) | 3 districts in Kampong province, Cambodia | 20 in-depth interviews 9 focus groups (87 recipients) | Low  No quotes provided | Barriers, ethical issues, social meaning, preconditions, scale up and sustainability |
| Jega, F.M., 2007 | Doctors and beneficiaries | Chiranjeevi scheme (vouchers for maternal health services) | 2 districts in Gujarat (mixed urban and rural), India | 52 interviews | Low  Representativeness of findings unclear  No indication of researchers’ impact on findings | Barriers, provider experiences, preconditions |
| Khan et al, 2010 | Recipients and their families, community health workers and community leaders | Janani Suraksha Yojana (payments to offset costs of access) | 24 villages in Uttar Pradesh (rural), India | 308 interviews | Moderate  Difficult to discern qualitative findings  No indication of ethical approval | Preconditions |
| Koehlmoos et al., 2008 | Structured interviews with recipients; in-depth interviews with community leaders, providers, and WHO; focus groups with voucher distributors and service providers | Maternal Health Voucher Scheme (vouchers for maternal health services) | 3 early implementation subdistricts (predominantly rural), Bangladesh | 31 structured interviews  26 in-depth interviews  12 focus groups | Low  No indication of researchers’ impact on findings | Barriers, provider experiences, ethical issues, social meaning, preconditions, scale up and sustainability |
| Krishna and Ananthpur, 2011 | Households in villages selected through two stage stratified sampling; pregnant women; focus groups with community leaders, health workers, NGO workers | Janani Suraksha Yojana (payments to offset costs of access) | Gulbarga and Raichur (rural) districts in Karnataka, India | Follow-up of 47 pregnant women  42 provider interviews  At least 12 focus groups | Low  No indication of researchers’ impact on findings | Barriers, provider experiences, ethical issues |
| Kumar et al., 2009 | Focus groups with recipients and with health workers | Janani Suraksha Yojana (payments to offset costs of access) | Una (predominantly rural) district in Himachal Pradesh, India | 5 focus groups with recipients  Unclear how many focus groups were with health workers | Moderate  No details on researchers or indication of their impact on the findings  No indication of ethical approval | Barriers, provider experiences, ethical issues, social meaning, preconditions |
| Kweku et al, 2007 | Interviews with health workers, retailers and pregnant women attending ANC | Volta voucher scheme (vouchers for merit goods) | Volta region (mixed urban and rural, Ghana | 957 interviews | Low  Difficult to discern source of findings | Barriers, preconditions |
| Lodh et al., 2009 | In-depth interviews with community health workers; focus groups with eligible women | Janani Suraksha Yojana (payments to offset costs of access) | Muzaffarpur district (predominantly rural) in Bihar, India | 43 interviews  4 focus groups | Moderate  No details on researchers or indication of their impact on the findings  No indication of ethical approval | Barriers, provider experiences, ethical issues, preconditions |
| Molyneux, M. and Thomson, M., 2011 | In-depth interviews with eligible women, programme managers, health workers and men; focus groups with women | Juntos and Bono Juana Azurduy programmes (conditional cash transfers) | Peru and Bolivia | 82 interviews  Focus groups with 159 women | Low  No details on researchers and their impact on the findings, or on questions asked  No indication of ethical approval | Barriers, ethical issues, social meaning |
| Mubyazi et al., 2010 | Pregnant women and mothers with infants | Tanzania National Voucher Scheme (voucher for merit goods) | Mkuranga and Mufindi districts (rural), Tanzania | 823 exit interviews  24 focus groups | Low  No indication of researchers’ impact on findings | Barriers, provider experiences, social meaning |
| Mushi et al., 2003 | Focus groups with community leaders, eligible women; in-depth interviews with health workers and retailers | Discount voucher scheme (voucher for merit goods) | Two districts (rural), Tanzania | 4 in-depth interviews  22 focus groups | Low  No indication of ethical approval  No consideration of researchers’ influence on data collected | Barriers, preconditions |
| Nandan et al, 2008 | Community leaders, health workers, programme managers, eligible women | Janani Suraksha Yojana (payments to offset costs of access) | 3 ‘priority’ districts in Orissa (predominantly rural), India | 240 semi-structured interviews  126 in-depth interviews  12 focus groups | Low  No consideration of researchers’ influence on data collected | Barriers, provider experiences, social meaning, preconditions |
| Nandan et al., 2008 | Community leaders, health workers, programme managers, eligible women | Janani Suraksha Yojana (payments to offset costs of access) | 3 districts in Orissa, India | 30 in-depth interviews  180 semi-structured interviews  6 focus groups | Low  No consideration of researchers’ influence on data collected | Barriers, preconditions |
| Nandan et al, 2010 | Programme managers, service providers, health workers and eligible women | MAMTA scheme (vouchers for maternal health services) | Delhi National Capital Territory, India | 262 in-depth interviews | Moderate  Limited description of interviews, e.g. length, language and interviewer  No indication of ethical approval | Barriers, provider experiences, ethical issues, preconditions,  scale up and sustainability |
| Pariyo et al, 2011 | Motorcycle riders who are contracted to transport pregnant women to health facilities | Makerere voucher scheme (vouchers for maternal health services) | Kamuli district, Uganda | 4 focus groups | Low  No details on researchers or consideration of their influence on findings | Barriers, preconditions |
| Powell-Jackson et al., 2009 | Interviews with providers and programme management; focus groups with health workers, NGO workers and programme managers | Safe Delivery Incentive Programme (payments to offset costs of access) | 10 districts, Nepal | 55 interviews  9 focus groups | Low  No consideration of researchers’ influence on data collected | Barriers, provider experiences, ethical issues, preconditions |
| Public Health Resource Network, 2010 | Village health nurses/auxiliary nurse midwives and anganwadi workers | Janani Suraksha Yojana (payments to offset costs of access) | 2 districts in Tamil Nadu, India | 55 in-depth interviews | Low  No indication of ethical approval  No consideration of researchers’ influence on data collected | Barriers, provider experiences, social meaning |
| Rai et al., 2012 | In-depth interviews community leaders; focus groups with communities | Janani Suraksha Yojana (payments to offset costs of access) | 12 villages in Jharkhand, India | 300 in-depth interviews  24 focus groups | Low  No consideration of researchers’ influence on data collected | Barriers, preconditions |
| Reproductive Health Vouchers Evaluation Team, 2011 | Interviews with service providers and clients; observations | Maternal Health Voucher Scheme (vouchers for maternal health services) | 22 sub-districts, Bangladesh | 1,100 exit interviews  295 provider interviews | Low  No consideration of researchers’ influence on data collected  No quotes provided | Preconditions |
| Reproductive Health Vouchers Evaluation Team, 2011 | Focus groups with communities and voucher distributors; in-depth interviews with providers and programme managers | Vouchers for Health (vouchers for maternal health services) | 6 rural districts and 2 informal settlements, Kenya | 1,823 exit interviews  201 provider interviews | Low  No consideration of researchers’ influence on data collected | Barriers, preconditions |
| Reproductive Health Vouchers Evaluation Team, 2012 | Exit interviews with clients in health facilities; interviews with providers | HealthyBaby vouchers (vouchers for maternal health services) | 30 health facilities, Uganda | 580 exit interviews  218 provider interviews | Low  No consideration of researchers’ influence on data collected  No quotes provided | Barriers, provider experiences, social meaning, preconditions |
| Rob et al., 2009 | Voucher users and non-voucher users | Pilot voucher scheme (vouchers for maternal health services) | Nabiganj subdistrict, Bangladesh | 30 semi-structured interviews | Low  No details on interviewers or possible influence on findings  No indication of ethical approval | Barriers, social meaning, precondition |
| Santhya et al., 2011 | Women eligible for JSY | Janani Suraksha Yojana (payments to offset costs of access) | Alwar and Jodhpur districts in Rajasthan, India | 48 in-depth interviews | Low  Difficult to discern qualitative findings  No consideration of researchers’ influence on data collected | Barriers, preconditions |
| Singh and Chaturvedi, 2007 | Interviews with community leaders, health workers and eligible women; focus groups with communities | Janani Suraksha Yojana (payments to offset costs of access) | Five districts in Uttar Pradesh and three districts in Uttarakhand, India | 50 focus groups  125 interviews | Moderate  No details on researchers or indication of their impact on the findings  No indication of ethical approval | Barriers, provider experiences, preconditions |
| Tami et al, 2006 | Focus groups with pregnant women and mothers; in-depth interviews with health staff | Discount voucher Scheme (voucher for merit goods) | Ulanga and Kilombero districts (rural), Tanzania | 5 focus groups  5 in-depth interviews | Low  No consideration of researchers’ influence on data collected | Barriers, preconditions, scale up and sustainability |
| Truzyan, N., 2010 | Focus groups with women; in-depth interviews with providers and programme managers | Obstetric Care State Certificate Program (vouchers for maternal health services) | Tavush, Ararat and Armavir districts (rural) and Yerevan (urban) in Armenia | 15 in-depth interviews  8 focus groups | Low  No consideration of researchers’ influence on data collected | Provider experiences, preconditions, scale up and sustainability |
| Uttekar et al., 2007 | Government officials, community leaders, NGOs, health workers and communities | Janani Suraksha Yojana (payments to offset costs of access) | 3 districts in Himachal Pradesh, India | 108 interviews | Low  Few details on interview methods, e.g. length and interviewer, or influence of researcher on findings | Barriers |
| Uttekar et al., 2007 | Government officials, community leaders, NGOs, health workers and community members | Janani Suraksha Yojana (payments to offset costs of access) | 3 districts in Rajasthan, India | 157 interviews | Low  Few details on interview methods, e.g. length and interviewer, or influence of researcher on findings | Barriers, preconditions |
| Uttekar et al., 2007 | Government officials, community leaders, NGOs, health workers and community members | Janani Suraksha Yojana (payments to offset costs of access) | 3 districts in Assam, India | 168 interviews | Low  Few details on interview methods e.g. length and interviewer | Barriers, preconditions |
| Uttekar et al., 2007 | Government officials, community leaders, NGOs, health workers and community members | Janani Suraksha Yojana (payments to offset costs of access) | 3 districts in Madhya Pradesh, India | 152 interviews | Low  Few details on interview methods, e.g. length and interviewer, or influence of researcher on findings | Barriers |
| Uttekar et al., 2007 | Government officials, community leaders, NGOs, health workers and community members | Janani Suraksha Yojana (payments to offset costs of access) | 3 districts in West Bengal, India | 107 interviews | Low  Few details on interview methods, e.g. length and interviewer, or influence of researcher on findings | Barriers |
| Uttekar et al., 2007 | Government officials, community leaders, NGOs, health workers and community members | Janani Suraksha Yojana (payments to offset costs of access) | 3 districts in Orissa, India | 114 interviews | Low  Few details on interview methods, e.g. length and interviewer, or influence of researcher on findings | Barriers |
| Uttekar et al., 2008 | Government officials, community leaders, NGOs, health workers and community members | Janani Suraksha Yojana (payments to offset costs of access) | 3 districts in Uttar Pradesh, India | 93 interviews | Low  Few details on interview methods, e.g. length and interviewer, or influence of researcher on findings | Barriers, preconditions |
| Uttekar et al., 2008 | Government officials, community leaders, NGOs, health workers and community members | Janani avam Bal Suraksha Yojana - Janani Suraksha Yojana in Bihar (payments to offset costs of access) | 3 districts in Bihar, India | 107 interviews | Low  Few details on interview methods, e.g. length and interviewer, or influence of researcher on findings | Barriers, preconditions |

Notes. ANC – antenatal care; BW – birth weight; CS – caesarean section; DFID – UK Department for International Development, ID – institutional delivery; ITN – insecticide-treated net; JSY – Janani Suraksha Yojana; MHVS – Maternal Health Voucher Scheme; NGO – non-governmental organisation; PNC – postnatal care; QoC – quality of care; SBA – skilled birth attendance, WHO – World Health Organization.
